# Retraction: Mast cells in skin scarring: a review of animal and human research

**DOI:** 10.3389/fimmu.2025.1720677

**Published:** 2025-10-16

**Authors:** Frontiers Editorial Office

The journal retracts the September 30–2020 article cited above.

Following publication, concerns were raised regarding the integrity of the images in the published figures. There were concerns about the images in Figure 2, which also appeared in another publication from a different publisher, but with different magnification and labelling. The authors failed to provide a satisfactory explanation during the investigation, which was conducted in accordance with Frontiers’ policies. As a result, the data and conclusions of the article have been deemed unreliable, and the article has been retracted.

The investigation followed Frontiers’ policies, and the retraction was approved by the Chief Executive Editor. The authors were informed about the retraction.

